# Safety and efficacy of GEMOX plus donafenib and tislelizumab as first‐line therapy for advanced epithelial malignant biliary tract cancer

**DOI:** 10.1002/cam4.5924

**Published:** 2023-04-11

**Authors:** Longrong Wang, Ning Zhang, Yixiu Wang, Ti Zhang, Weiping Zhu, Anrong Mao, Yiming Zhao, Lu Wang

**Affiliations:** ^1^ Department of Hepatic Surgery, Fudan University Shanghai Cancer Center Department of Oncology, Shanghai Medical College, Fudan University Shanghai China

**Keywords:** biliary tract cancer, donafenib, first‐line, GEMOX, tislelizumab

## Abstract

**Aim:**

This study was aimed to evaluate the safety and the efficacy of gemcitabine and oxaliplatin (GEMOX) combined with donafenib plus tislelizumab as the first‐line treatment for patients with unresectable biliary tract cancer (BTC).

**Methods:**

This is a prospective single‐center exploratory study. Eligible patients (Stage III/IV BTC, at least one measurable disease according to RECIST v1.1, etc.) received gemcitabine 1000 mg/m^2^ IV Q3W, oxaliplatin 100 mg/m^2^ IV Q3W, donafenib 200 mg PO BID, and tislelizumab 200 mg IV Q3W until disease progression, unacceptable toxicity, or withdrawal of consent whichever occurred first. The primary endpoint was safety and secondary endpoints included disease control rate (DCR), objective response rate (ORR), conversion rate, and overall survival (OS).

**Results:**

A total of 13 patients were enrolled. The median follow‐up time was 420 days (range 345–487). The median duration of treatment was four cycles (range 1–15). The incidence of ≥Grade 3 treatment‐related adverse events (TRAEs) was 53.8% and no Grade 5 TRAE. The most frequent Grade 3–4 TRAEs were rash (4/13, 30.8%), platelet count decreased (2/13, 15.4%), and fatigue (2/13, 15.4%). Tumor response was assessed in eight evaluable patients; ORR was 25.0% (95% CI, 3.2%–65.1%) and DCR 87.5% (95% CI, 47.3%–99.7%). The median PFS was 4.8 months (95% CI, 1.25‐NE). Three Stage III patients underwent subsequent surgery with a conversion rate of 23.1%. The median OS was not estimable.

**Conclusions:**

GEMOX combined with donafenib plus tislelizumab as the first‐line therapy for unresectable BTC showed manageable toxicity and encouraging efficacy especially in terms of promising conversion rate in Stage III patients.

## 1 INTRODUCTION

Biliary tract cancers (BTC), including gallbladder cancer and cholangiocarcinoma, are rare malignancies with a rising incidence.^1^ Nearly 70% of the newly diagnosed patients are in advanced stage,^2^ and hardly have an opportunity to undergo curative surgical resection. Even though receiving radical surgery, recurrence is common in patients with early‐stage disease.^3^ Currently, gemcitabine based systemic chemotherapy is the standard first‐line therapy and the most commonly used treatment for patients with unresectable BTC^4^, ^5^ The efficacy of chemotherapy is limited. The median overall survival (OS) is less than 12 months and prognosis is poor. New therapeutic drugs and combinations need to be developed.

Immunotherapy and targeted therapy may expand treatment options for BTC, as with other solid tumors. Researchers have explored using these therapies alone or in combination with the standard of care treatment to improve efficacy.^6^

Immunotherapy represented by immune checkpoint inhibitors (ICIs) as monotherapy has shown limited antitumor activity in unselected patients with advanced BTC, though surprising efficacy was achieved in selected patients.^6^, ^7^ For example, in the KEYNOTE‐158 and KEYNOTE‐028 studies, previously treated patients with advanced BTC, regardless of PD‐L1 expression, received pembrolizumab monotherapy and the objective response rate (ORR) was only 5.8%–13.0%, median progression‐free survival (PFS) was 1.8–2.0 months and median OS was 5.7–7.4 months, while a small group of patients with high levels of microsatellite‐instability high /defective mismatch repair (dMMR) in KEYNOTE‐158 achieved an ORR of 40.9%, median PFS of 4.2 months and median OS of 24.3 months.^8^, ^9^ ICI combination therapies were also investigated. Comparing to ICI monotherapy, combining ICIs with other non‐ICI treatments may be a better choice.^10^ Several ongoing trials are evaluating ICIs plus chemotherapy as first‐line treatment in advanced BTC and recently the results of the phase III TOPAZ‐1 and KEYNOTE‐966 trials suggest that combining ICIs and chemotherapy may improve OS and PFS compared to chemotherapy alone.^6^, ^11^, ^12^, ^13^

Targeted agents, including the epithelial growth factor receptor inhibitors, fibroblast growth factor receptor inhibitors and anti‐angiogenic agents, were tested in combination with standard chemotherapy in attempts to improve clinical outcomes but have not demonstrated positive results so far.^6^, ^14^, ^15^, ^16^, ^17^, ^18^, ^19^, ^20^, ^21^, ^22^ However, the combination of targeted therapies and ICIs have shown promising efficacy in second‐line treatment for BTC. Lenvatinib, a tyrosine kinase inhibitor, in combination with PD‐1 resulted in an ORR of 9.7%–30.4% and disease control rate (DCR) of 67.7%–85.7% in pretreated patients with advanced BTC.^23^, ^24^ Even more remarkably, reported at the EMSO Virtual Congress 2020, toripalimab plus lenvatinib in combination with oxaliplatin and gemcitabine (GEMOX) as first‐line treatment in advanced and unresectable intrahepatic cholangiocarcinoma showed an ORR of 80%, a DCR of 93.3%, and a 6 month OS rate of 90% in a single‐arm phase II study from a Chinese center; and 43% of patients experienced Grade 3 or higher adverse events.^25^ Although chemotherapy combined with tyrosine kinase inhibitors and immune checkpoint inhibitors in the treatment of BTC has shown amazing efficacy, the drug‐related toxicities and safety issue associated with the combination of four drugs warrants further exploration. Meanwhile, how to further optimize the scheme while giving consideration to safety is still challenging.

Donafenib, which is newly approved in June 2021 for the treatment of patients with hepatocellular carcinoma (HCC) who have no prior systemic antitumor therapy, is an oral small molecule multikinase inhibitor of multiple receptor kinases, including vascular endothelial growth factor receptor, platelet‐derived growth factor receptor and Raf kinases, thus suppressing tumor cell proliferation and angiogenesis. In the phase II/III trial, donafenib demonstrated a significantly prolonged OS over sorafenib and favorable safety and tolerability in patients with unresectable or metastatic HCC.^26^ Recently, donafenib in combination with PD‐1 inhibitors has showed promising results in a few clinical studies.^27^, ^28^

The purpose of this study was to evaluate the safety and efficacy of gemcitabine and oxaliplatin (GEMOX) combined with donafenib plus tislelizumab used as first‐line treatment in patients with advanced epithelial malignant BTC.

## 2 METHODS

### 2.1 Study design and patients

This is a prospective single‐center exploratory study registered on clinicaltrials.gov (NCT04979663). Eligible patients were aged of 18–80 years (inclusive) with histologically or cytologically documented Stage III/IV (American Joint Committee on Cancer Staging Manual, 8th Edition)^29^ epithelial malignancies arising from the biliary tree, at least one measurable disease according to RECIST v1.1, Eastern Cooperative Oncology Group (ECOG) performance status (PS) of 0–1, good function of major organs, life expectancy of at least 3 months, and being naive to any anti‐BTC treatment. Patients with long‐term unhealed wound or bone fracture, organ transplant history, abnormal coagulation function, or had thrombosis event attack (e.g., cerebrovascular accident, deep venous thrombosis, pulmonary embolism) within 1 year before enrollment, or with immune deficiency were excluded.

The study protocol was approved by the Ethics Committee of Fudan University Shanghai Cancer Center (approval number 2102231‐12) and the study conduct followed the Declaration of Helsinki, Good Clinical Practice Guidelines and Chinese regulatory requirements. Written informed consent was obtained from every patient.

### 2.2 Treatment

Eligible patients received gemcitabine 1000 mg/m^2^ IV Q3W, oxaliplatin 100 mg/m^2^ IV Q3W, donafenib 200 mg PO BID, and tislelizumab 200 mg IV Q3W until successfully converted to surgical treatment, disease progression, unacceptable toxicity or withdrawal of consent, whichever occurred first.

Dose modifications of donafenib and tislelizumab were not permitted. In case of intolerable adverse reaction only related to donafenib or tislelizumab, study treatment was to be discontinued. For adverse events related to chemotherapy, the dose of gemcitabine/oxaliplatin was allowed to reduce by 25% at a time. A dose increase of gemcitabine/oxaliplatin was not permitted. Oxaliplatin was to be stopped permanently if any Grade 3 or worse adverse reaction occurred in cycle 3 or later, and these patients then received gemcitabine alone with donafenib plus tislelizumab.

### 2.3 Procedures

Safety monitoring included a complete blood count (CBC) once a week and liver and kidney function tests, biochemistry, coagulation, thyroid function, 12‐lead electrocardiogram and myocardial enzymes once every 3 week. Vital signs and surgical feasibility evaluation were performed every two treatment cycles.

Adverse events were collected from the date of written informed consent until 30 days after the last study dosing.

Tumor response was assessed at two‐cycle intervals based on enhanced magnetic resonance imaging or computerized tomography as per RECIST v1.1.

### 2.4 Study endpoints

The primary endpoint was safety, which included the incidence of adverse events and serious adverse events. Adverse events were evaluated according to the common drug toxicity classification standard (NCI‐CTCAE 4.0) specified by the National Cancer Institute of the United States. Secondary endpoints included DCR, which is defined as the total number of patients assessed as complete response (CR), partial response (PR), and stable disease (SD) accounted for the total number of patients; ORR, the total number of patients assessed as CR or PR accounted for the total number of patients; PFS, the time from first date of study dosing to disease progression or death from any cause, whichever occurred first; OS, the time from first date of study dosing to death from any cause and conversion rate, the proportion of patients who received radical surgical treatment with negative pathological margins.

### 2.5 Statistical analyses

The safety endpoints were assessed in safety population, which included patients who received at least one dose of study drugs and had any item of safety data available. The efficacy analyses were performed in evaluable analysis population, which consisted of patients who received at least one dose of study drugs and had best overall response assessment. The last observation carried forward method was used for handling the missing visit values.

The number of patients and the proportion with events of interest were provided for categorical data and median with interquartile or range were used for continuous data, as appropriate. The ORR and DCR were presented with point estimate and its 95% confidence intervals (CI) calculated using the Clopper‐Pearson method. Time to event endpoints including PFS and OS were analyzed by using Kaplan–Meier method, with a 95% CI estimated on the basis of the Brookmeyer Crowley method. All statistical analyses were conducted using SAS® version 9.4 (SAS Institute).

## 3 RESULTS

### 3.1 Subject characteristics

A total of 13 eligible patients were enrolled from March 2021 to August 2021, including 5 males and 8 females; aged of 53–72 years; four Stage III and nine Stage IV; all ECOG PS of 1; four gallbladder carcinoma, two hilar cholangiocarcinoma and seven intrahepatic cholangiocarcinoma. As of the data cutoff date (July 14, 2022), all patients discontinued treatment including three patients who underwent surgery. The other reasons for discontinuation were disease progression, withdrawal of consent and unable to continue treatment because of the travel restrictions due to the COVID‐19 pandemic. A median number of 4 cycles (range 1–15) of study treatment was received and the median treatment duration of donafenib was 87 days (range 17–317). The median follow‐up time was 420 days (range 345–487) (Table 1).

**TABLE 1:** Baseline characteristics.

	No. of patients (%) (*N* = 13)
Age (years), median (range)	65.0 (53‐72)
Sex, *n* (%)	
Male	5 (38.5%)
Female	8 (61.5%)
Tumor type, *n* (%)	
Gallbladder carcinoma	4 (30.8%)
Hilar cholangiocarcinoma	2 (15.4%)
Intrahepatic cholangiocarcinoma	7 (53.8%)
AJCC stage, *n* (%)	
Stage III	4 (30.8%)
Stage IV	9 (69.2%)
ECOG PS score, *n* (%)	
1	13 (100%)
Pain score, *n* (%)	
0	10 (76.9%)
2	2 (15.4%)
4	1 (7.7%)
Tumor marker (*n* = 11), median (IQR)	
CA‐199 (U/mL)	125.00 (4.52–1000.01)
AFP (ng/mL)	3.48 (1.80–11.40)
CEA (ng/mL)	6.33 (1.56–12.80)
# of treatment cycle, median (range)	4.0 (1–15)
Duration of donafenib treatment (days), median (range)	87.0 (17–317)
Follow‐up time (days), median (range)	420 (345–487)

Abbreviations: AFP, alpha fetal protein; AJCC, American Joint Committee on Cancer; CA, carbohydrate antigen; CEA, carcinoembryonic antigen; ECOG PS, Eastern Cooperative Oncology Group performance status; IQR, interquartile range.

### 3.2 Safety

Treatment‐related adverse events (TRAEs) occurred among all patients (100%), including seven (53.8%) patients who had Grade 3 TRAEs, and one (7.7%) patient who had a Grade 4 TRAE (Table 2). The incidence of Grade 3 or higher TRAEs was 53.8% (one patient had both Grade 3 and Grade 4 TRAE). No Grade 5 TRAE was reported. The most frequently reported TRAEs were rash (8/13 [61.5%]; Grade 3–4 in 4 [30.8%] patients), platelet count decreased (8/13 [61.5%]; Grade 3–4 in 2 [15.4%] patients), anemia (7/13 [53.8%]; no Grade 3–4), pyrexia (7/13 [53.8%]; no Grade 3–4) and fatigue (6/13 [46.2%]; Grade 3–4 in 2 [15.4%] patients) (Table 3).

**TABLE 2:** Adverse event summary.

	No. of patients (%) (*N* = 13)
At least one TEAE	13 (100%)
Grade 3	7 (53.8%)
Grade 4	1 (7.7%)
≥Grade 3	7 (53.8%)
At least one TRAE	13 (100%)
Grade 3	7 (53.8%)
Grade 4	1 (7.7%)
≥Grade 3	7 (53.8%)

Abbreviations: TEAE, treatment‐emergent adverse event; TRAE, treatment‐related adverse event.

**TABLE 3:** Common treatment‐related adverse events (≥2 patients).

TRAE	No. of patients (%)	
	All grades	≥Grade 3
Rash	8 (61.5%)	4 (30.8%)
Platelet count decreased	8 (61.5%)	2 (15.4%)
Anemia	7 (53.8%)	0
Pyrexia	7 (53.8%)	0
Fatigue	6 (46.2%)	2 (15.4%)
Vomiting	4 (30.8%)	0
Palmar‐plantar erythrodysaesthesia syndrome	3 (23.1%)	0
White blood cell count decreased	3 (23.1%)	0
Neutrophil count decreased	3 (23.1%)	0
Alanine aminotransferase increased	2 (15.4%)	0
Chills	2 (15.4%)	0
Proteinuria	2 (15.4%)	0

Abbreviation: TRAE, treatment related adverse event.

### 3.3 Efficacy

**FIGURE 1:**
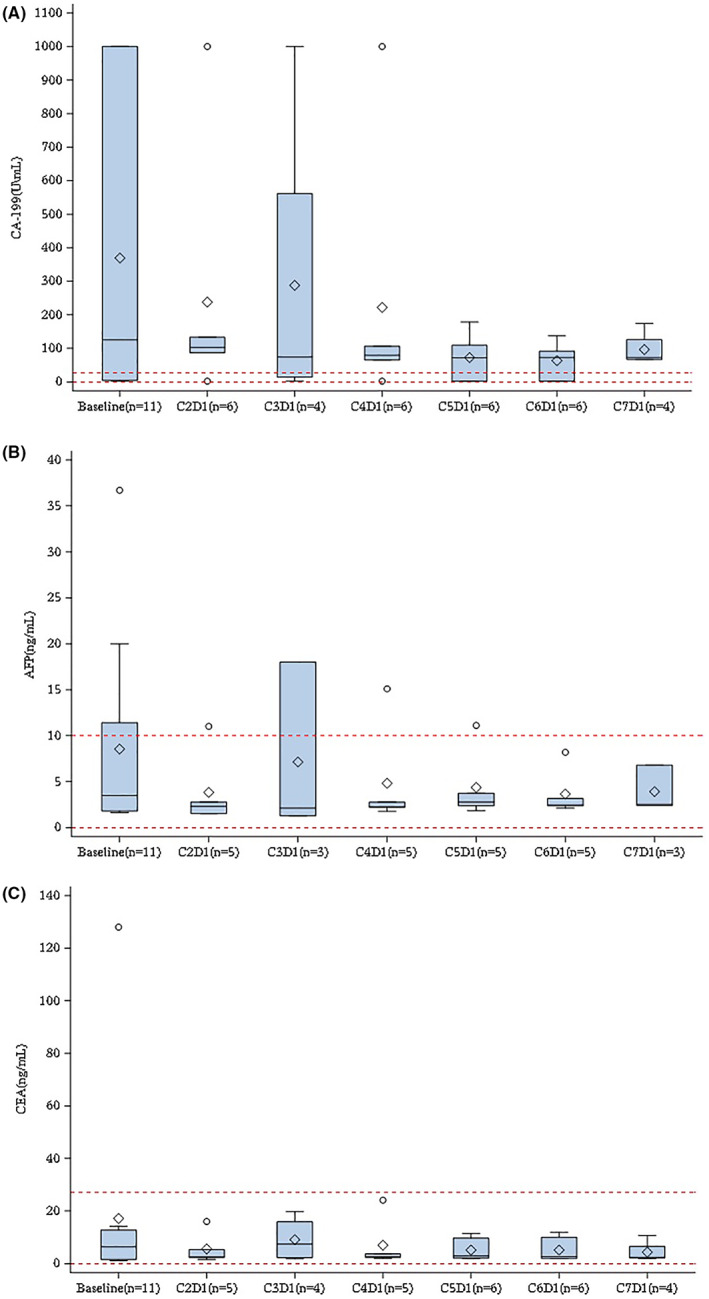
(A) CA‐199 box plot at specified visits. (B) AFP box plot at specified visits. (C) CEA box plot at specified visits. AFP, alpha fetal protein; CA, carbohydrate antigen; CEA, carcinoembryonic antigen.ο‐extreme value; solid line in the box‐Median; ◇‐Mean; red dotted line‐normal range

The median (interquartile range, IQR) levels of carbohydrate antigen (CA)‐199, alpha fetal protein and carcinoembryonic antigen at baseline were 125 U/mL (4.52–1000.01), 3.48 ng/mL (1.8–11.4) and 6.33 ng/mL (1.56–12.80), respectively. These biomarker values at different visits are presented in Figure 1A–C. Of them, CA‐199 showed a decreasing trend when compared to baseline.

**FIGURE 2:**
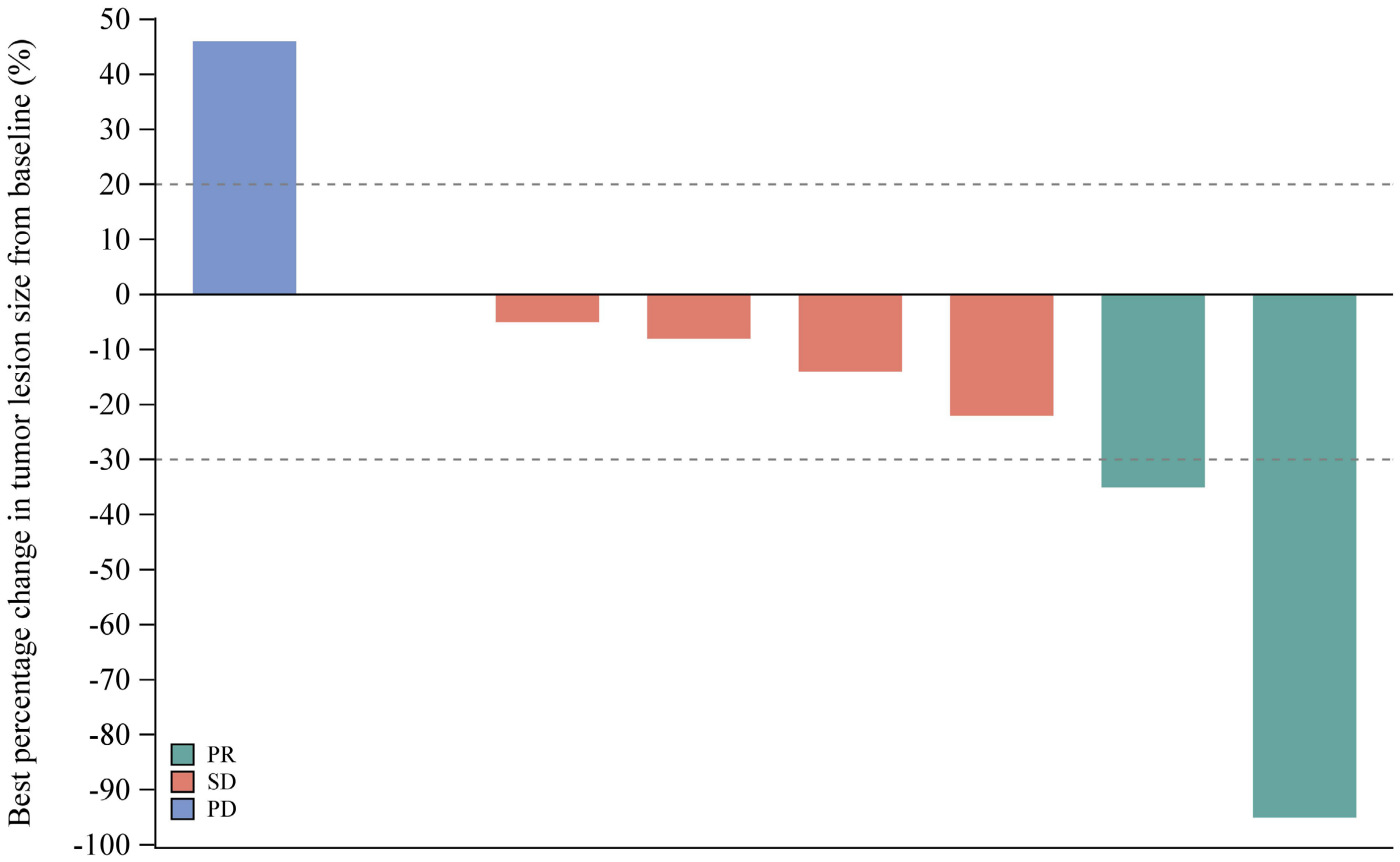
Waterfall plot of best percentage change in tumor lesion size from baseline per RECIST v1.1.

Best overall response was evaluable for eight patients, among whom two PR (25.0%), five SD (62.5%) and one PD (12.5%), resulting in an ORR of 25.0% (95% CI, 3.2%–65.1%) and DCR of 87.5% (95% CI, 47.3%–99.7%) (Table 4). The best percentage change in the size of target lesions per patient is shown in Figure 2.

**TABLE 4:** Tumor response per RECIST v1.1 in evaluable patients.

	No. of evaluable patients (%) (*N* = 8)
Best overall response, *n* (%)	
PR	2 (25.0%)
SD	5 (62.5%)
PD	1 (12.5%)
Objective response rate (CR + PR), *n* (%)	2 (25.0%)
95% CI	3.2%–65.1%
Disease control rate (CR + PR + SD), *n* (%)	7 (87.5%)
95% CI	47.3% to 99.7%

Abbreviations: CI, confidence interval; CR, complete response; PD, progressive disease; PR, partial response; SD, stable disease.

**FIGURE 3:**
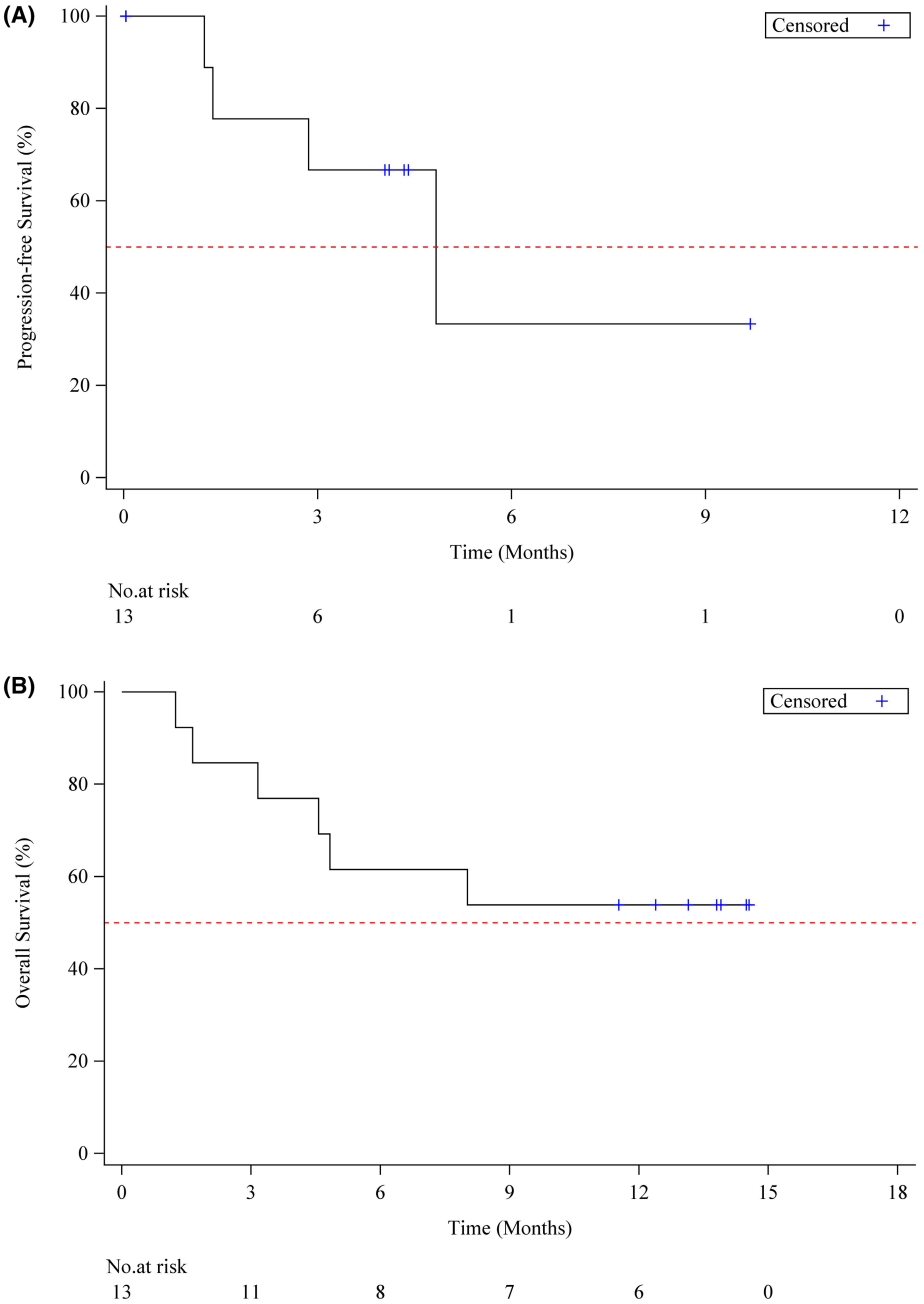
(A) Kaplan–Meier estimates of progression‐free survival. (B) Kaplan–Meier estimates of overall survival.

Median PFS was 4.8 months (95% CI, 1.25‐NE). The probability of PFS at 6 months was 33.3% (95% CI, 1.6%–74.8%) (Figure 3A). The median OS was not estimable. The 12 month survival probability was 53.8% (95% CI, 24.8%–76%) (Figure 3B).

Three Stage III patients underwent subsequent surgery with a conversion rate of 23.1%.

## 4 DISCUSSION

In recent 10 years, the first‐line treatment of advanced BTC has progressed slowly. TKI combined with PD‐1/PD‐L1 monoclonal antibody showed significant effectiveness in multiple tumors except BTC. Clinical investigators have been exploring combination therapy for advanced BTC with the aim of maximizing the response and minimizing the side effects. Based on the TOPAZ‐1 trial, on September 2, 2022, FDA approved duvalizumab combined with gemcitabine and cisplatin as first‐line for the treatment of patients with locally advanced or metastatic BTC.^11^

This study is another attempt to investigate the quadruple therapy with different mechanisms of action as the first‐line treatment for unresectable BTC and obtained promising results. To our knowledge, this is the first study to use this combination as first‐line treatment.

The pattern of adverse event was as anticipated and consistent with the earlier reports regarding immunotherapy and/or targeted drugs in combination with chemotherapy. The most frequent drug‐related adverse events were hematological toxicities except rash. These hematological toxicities were known to be associated with gemcitabine and oxaliplatin,^30^ while rash occurred more often with combined therapies.^11^, ^18^, ^31^

Targeted drugs can induce rapid tumor death, lead to the release of new antigens, enhance immune response, and thus enhance the effect of ICI treatment; on the other hand, ICI therapy can consolidate the antitumor effect of targeted therapy.^32^ Chemotherapy may weaken the immunosuppressive effect of microenvironment, increase the cross presentation of tumor antigens, support the penetration of immune cells into the tumor core, and also strengthen the immune system by increasing the proportion of cytotoxic lymphocytes and regulatory T cells and the number of antigen presenting cells.^33^ Therefore, the combination of target and immunotherapy and chemotherapy can theoretically improve the treatment response.

In this study, CA199 median showed an obvious downward trend from baseline. Reduction in biomarker CA199 is associated with treatment response.^34^ The DCR of 87.5%, ORR of 25.0%, and median PFS of 4.8 months seemed encouraging since the majority of patients had Stage IV disease at entry. However, the antitumor activity of this quadruple therapy needs to be verified in phase II and III trials.

Surgery is the cornerstone of curative therapy. For some local unresectable patients, resection may be feasible due to lesion shrinkage resulted from systemic therapy. This is also what the researchers’ efforts aiming at. In this study, tumor burden reductions from baseline occurred in six patients (46.1%), with highest reduction of 95%. Notably, 3 of 4 Stage III patients met surgical criteria based on investigator's judgment and subsequently underwent surgery. Pathology confirmed R0 resection thus conversion rate was 23.1%. Of the three patients, two were intrahepatic cholangiocarcinoma and one perihilar cholangiocarcinoma. One was cTMN Stage T3 N0 M0 and two T2 N1 M0. One received six treatment cycles before surgery and two received 7 cycles. The best overall response was SD for two and PR for one. Study reported a conversion rate of 6.7% (2/30) with toripalimab + lenvatinib + GEMOX^8^ and 34.2%(13/38) with levatinib + pembrolizumab.^35^

There are some limitations of this study. This is a phase I investigational study initiated by the investigator and was carried out in the context of clinical practice. Its sample size was not based on statistical assumption but was on available subjects. Only 13 Chinese patients were enrolled. At the same time, due to the impact of the epidemic situation of the COVID‐19, some patients could not return to the site for follow‐up in time, resulting in that only eight patients were evaluable for efficacy. Secondly, the patients are from one medical institute, which may cause bias when extrapolating study results to large population. Lastly, these patients were with varied types of BTC and tumor location may be a factor predicting response to systemic therapy.^36^ Further study with large population is warranted for tumor location sub‐analysis.

Looking ahead, efforts may focus on identifying reliable biomarkers of response to ICIs and targeted agents in BTC, clarifying the role of PD‐L1 expression, microsatellite instability, mismatch repair, tumor mutational burden, DNA damage repair alterations, and other emerging predictors.^7^, ^12^

In conclusion, GEMOX combined with donafenib plus tislelizumab as the first‐line therapy for locally advanced or metastatic BTC showed manageable toxicity and encouraging efficacy especially in terms of a promising conversion rate in Stage III patients. For some patients with unresectable or potentially resectable BTC, the conversion rate of 23.1% is of reference significance. A phase II study using this treatment scheme is now in progress and that further study with large sample size is expected to open up new ideas for BTC conversion therapy/neoadjuvant therapy.

## AUTHOR CONTRIBUTIONS

**Longrong Wang:** Investigation (equal); writing – original draft (lead). **Ning Zhang:** Investigation (supporting); writing – original draft (supporting). **YiXiu Wang:** Investigation (equal); writing – review and editing (supporting). **Ti Zhang:** Investigation (equal); writing – review and editing (supporting). **Weiping Zhu:** Investigation (equal); writing – review and editing (supporting). **Anrong Mao:** Investigation (equal); writing – review and editing (supporting). **Yiming Zhao:** Investigation (equal); writing – review and editing (equal). **Lu Wang:** Conceptualization (lead); writing – review and editing (lead).

## FUNDING INFORMATION

This study was supported by grants from Beijing iGandan Foundation (HYXH202042, HYXH2021074).

## CONFLICT OF INTEREST STATEMENT

The authors declare that there are no conflicts of interest.

## Data Availability

The data that support the findings of this study are available from the corresponding author upon reasonable request.
